# Exploring service users’ views to reduce inequalities in healthcare

**DOI:** 10.1111/hex.12836

**Published:** 2018-10-14

**Authors:** Louise Condon

**Affiliations:** ^1^ Professor of Nursing at Swansea University and Abertawe Bro Morgannwg University Health Board

The UK has recently celebrated the 70th birthday of the National Health Service, and there has been much reflection on changes in health care since 1948, and focus on how the NHS is currently performing in relation to the health services of other countries. Comparing across health systems is notoriously difficult, even from the perspective of deciding which indicators to measure.^1^ Simply comparing health care expenditure does not suffice to represent the quality of a health system,^2^ although this measure continues to be widely reported. In order to make comparison across health systems the WHO has considered the following: overall level of population health, health inequalities, health system responsiveness (and how this is distributed across the population) and how the financial burden is spread. These give a high‐level picture of the health service delivered in each participating country, with clear recognition of public health values.

However, for those who use health services the focus is often not on the quality of the health service overall, but on their own personal experience of the care received. If we think of our own experience as service users, what matters most is often the way things are done, how we are treated by those providing care, the extent to which our autonomy is preserved and the relationships we form with service providers. By virtue of its commitment to illuminating the service user's voice, Health Expectations is uniquely well placed to attract and publish high‐quality research which explores the experience of care at a granular level. Seeking complementary and alternative care outside the health system is increasingly common (see Chou et al.), but at some points of life people inevitably become mainstream health system service users. One possibly unexpected consequence of becoming a service user may be becoming the focus of professional assessment and judgment. Lauridsen et al. tracked the experiences of women who were categorized as being overweight or obese in pregnancy and assessed the impact over time. Understanding more of what it is like to experience treatment and care is important for those who commission and deliver health services, in order to improve the effectiveness of interventions.

Sudden illness also precipitates health service use, and Perry et al. assessed the impact of newly introduced centralized stroke care pathways from the perspective of patients. Mortality from ischaemic stroke is higher in the UK than in comparable countries,^3^ and care in a specialist stroke unit has been recommended as the biggest single factor to improve clinical outcomes. When stroke services were redesigned in two cities in the England, Perry et al. explored the experiences of patients (and their relatives) who followed the new centralized pathways. They found that the disadvantages of travelling further for care were outweighed for most by the opportunity to receive the best quality care. Additionally, receiving clear and accessible information from service providers was key in maximizing patients’ experience of care. Exploring views is recognized as important in improving the quality of health services, but there can be uncertainty about how best to respond to such feedback. Based on patients’ stories about care received from adult mental health services, Baines et al. have developed a conceptual framework to guide effective responses to service user feedback.

Good communication (even down to the level of the words used to speak to service users) is not just the “icing on the cake” but affects health outcomes at a profound level (Stans et al., Reilly et al.). Health inequalities have a profound effect upon experiences of using health services and people who are less advantaged for physical, psychological or social reasons have an even greater need for thoughtful and appropriate care. How to ensure protection for vulnerable groups was one of the knotty problems Swiss citizens considered when asked to reach a consensus on health care allocation decisions (Schindler et al.). The extent to which personal responsibility plays a part in coverage for all was a contentious issue, but the authors conclude that participants were able to set priorities for complex health issues, making “trade‐offs” in the process.

This edition of Health Expectations shows that in‐depth exploration of service users’ experience is fundamental in shaping health services to be more accessible and acceptable for service users, hence increasing their effectiveness. Improving the experience of service use for the most vulnerable has a major part to play in reducing inequalities in health.
